# Development and evaluation of reverse transcription-loop-mediated isothermal amplification assay for rapid detection of enterovirus 71

**DOI:** 10.1186/1471-2334-11-197

**Published:** 2011-07-18

**Authors:** Weifeng Shi, Kun Li, Yun Ji, Qingbo Jiang, Mei Shi, Zuhuang Mi

**Affiliations:** 1 Department of Clinical Laboratory, The Third Affiliated Hospital of Suzhou University, No. 185 Juqian street, Changzhou, Jiangsu 213003, P. R. China; 2 Wuxi Clone Gen-Tech Institute, No. 26 Fenghua Road, Wuxi, Jiangsu 214026, P. R. China

## Abstract

**Background:**

Hand, foot, and mouth disease (HFMD) caused by enterovirus 71 (EV71) is very common in China. It is difficult to distinguish between EV71 and coxsackievirus A16 (CVA16) infections in clinical HFMD patients. Routine laboratory diagnosis of EV71 infection is time-consuming and requires expensive instruments. In this study, we have developed a one-step, single tube, reverse transcription-loop-mediated isothermal amplification (RT-LAMP) assay for rapid and sensitive detection of EV71.

**Methods:**

Six primers that can recognize 6 distinct regions on the VP2 gene of EV71 were designed for RT-LAMP assay. The amplification was completed by incubating all reagents in a single tube with reverse transcriptase and *Bst *DNA polymerase under the isothermal condition (60°C) for 60 min, and could be evaluated by using GoldView staining under a handheld ultraviolet torch lamp or electrophoresis analysis.

**Results:**

A total of 123 specimens collected from suspicious patients with HFMD were simultaneously detected by RT-LAMP and PCR fluorescence probing assay. The RT-LAMP amplified products containing EV71 were digested by HinfI and TaqI restriction endonucleases; in contrast, non-specific products with CVA16, coxsackievirus A4 and coxsackievirus B3 could not be detected in RT-LAMP assay. Meanwhile, RT-LAMP assay could amplify EV71 virus with a detection limit of 1 PFU/ml within 60 min. Compared with PCR fluorescence probing assay, RT-LAMP assay exhibited 98.4% identity during the detection of EV71 viral RNA without the missing of positive samples.

**Conclusion:**

Our results indicated that RT-LAMP is a rapid, sensitive, specific and accurate method for the detection of EV71 in clinical specimens. Therefore, this developed method has potential application for rapid and comprehensive surveillance for EV71 infection, especially in developing country.

## Background

HFMD, a common illness in children, can be caused by many human enteroviruses such as coxsackie viruses A4, A5, A6, A10, A16, B1, B3, and EV71 [1-4]. Among these viruses, human EV71 and CVA16 are major causative agents of HFMD. EV71 and CVA16 infections in HFMD are indistinguishable. However, EV71 infection is frequently associated with serious neurological complications and fatalities [5]. EV71 was initially isolated from the stool of a 9-month-old infant with fatal encephalitis in California in 1969 [6]. Subsequently, the prevalence of EV71 infection has been reported in many countries and regions, such as Taiwan, Hongkong, Malaysia and Singapore, as well as Guangdong, Hunan, Jiangsu and Fuyang in China [7-13]. EV71 and CVA16 infections mainly occurred in children under 5 years old. However, patients infected with EV71 are liable to cause aseptic meningitis, encephalomyelitis, and pulmonary edema [14,15].

Traditional EV71 infection is primarily dependent on serodiagnosis, and virus culture and identification. However, these methods are time-consuming and easy to produce cross-immune response with CVA16. Recently, reverse transcription-PCR (RT-PCR) and real-time PCR assays have been used for EV71 detection [16-18]. These nucleic acid amplification methods with intrinsic disadvantages of requiring sophisticated instrumentations and expensive reagents may not be the best choice for basic clinical settings in developing countries or in field situations. Therefore, it is necessary to develop a rapid, reliable, and simple molecular test to take the place of existing techniques. In 2000, a newly developed LAMP method with the characteristics of simplicity, rapidity, specificity, and cost-effectiveness has the potential to replace PCR [19]. LAMP is based on the principle of strand displacement reaction so that the stem-loop structure can amplify the target with high specificity, selectivity and rapidity under isothermal conditions. LAMP can also produce a large amount of target DNA and by-product magnesium pyrophosphate for the formation of turbidity. Therefore, LAMP assays have been widely used to detect a variety of infectious diseases, such as bacterial, fungus and viral infections [20-22].

Nowadays, the application of LAMP method in EV71 detection was less reported [23]. In the present study, we have developed a one-step, single-tube, and real-time RT-LAMP assay to detect EV71. The amplified products can be stained by double-stranded DNA binding fluorescent dye (GoldView stain), and observed through naked eyes under the UV lamp. Compared with PCR fluorescence probing assay, RT-LAMP had high sensitivity and specificity during EV71 detection, which is suitable for the application in primary health care agencies.

## Methods

### Specimen collection

A total of 123 suspicious patients with HFMD under 5 years old were enrolled in 2009 in Changzhou, China. This study was approved by the local ethics committee and all patients were provided a written informed consent. Totally 93 pharyngeal swabs, 20 vesicular fluid swabs and 10 fecal samples were collected from these patients within 4 days after the onset of infection, and stored in 3-5 ml of preservation solution (Hanks solution containing 10 μg/ml gentamicin and 0.25 μg/ml amphotericin B) at -70°C for analysis.

### Virus isolation and identification

Specimens were inoculated into rhabdomyosarcoma (RD) cells for the isolation of EV71. Enterovirus strains were identified by using immunofluorescence test with EV71 monoclonal antibody (Chemicon International Inc.).

### Primer design for RT-LAMP

**Figure 1:**
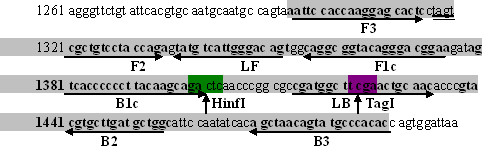
**Sequences of RT-LAMP primers for EV71**.

Six primers were designed according to the VP2 gene sequences of EV71 virus published in GenBank (accession No. AY465356), and was aligned with available VP2 gene sequences of other strains, including the circulating strains in China responsible for recent epidemics, to identify the conserved regions using DNASIS software. The potential target region of 194 bp corresponding to the genome positions from 1296 to 1489 was selected from the aligned sequences, including two inner, two outer primers and two loop primers (Figure 1). Two inner primers were called the forward inner primer (FIP) and the backward inner primer (BIP), which contained two distinct sequences corresponding to the sense and antisense sequences of the target DNA, for priming in the first stage and self-priming in later stage, respectively. FIP contained the sequence (F1c) complementary to F1 and F2. BIP contained B1 and the sequence (B2c) complementary to B2. Two outer primers were composed of F3 and the sequence (B3c) complementary to B3 (Table 1).

**Table 1:** The RT-LAMP primers

Primer names	Length of oligonucleotides (bp)	Primer sequences
F3	20	5'-AATTCCACCAAGGAGCACTC-3'

B3	20	5'-GTGTGGGCATACTGTTAGCT-3'

FIP	40	5'-TTCCGTCCCTGTACCGCCTGCTAGTCGCTGTCCTACCAGA-3'

BIP	40	5'-GTCACCCCCCTTACAAGCAGAGCCAGCATCAAGCACGTAC-3'

LF	17	5'-ACTGTCCCAATGACATA-3'

LB	20	5'-CGATGGCTTCGAACTGCAAC-3'

### Extraction of viral RNA

Prior to RNA extraction, fecal samples were treated with chloroform according to the protocol described elsewhere [24]. The genomic viral RNA was extracted from 100 μl of swab specimens or pretreated feces by using the QIAamp viral RNA extraction kit (Qiagen, Germany) according to the manufacturer's instructions. The RNA was eluted from the QIAspin columns in a final volume of 100 μl of elution buffer and kept at -70°C until further analysis.

### Reaction system of RT-LAMP

The RT-LAMP reaction was performed in a 25 μl of reaction mixture containing 0.8 μM (each) FIP and BIP inner primers, 0.2 μM forward outer primer (F3) and backward outer primer (B3), 0.4 μM LF and LB (each), 0.4 mM deoxynucleoside triphosphates (dNTPs), 1 M betaine (Sigma, USA), 20 mM Tris-HCl (pH 8.8), 10 mM KCl, 10 mM (NH_4_)_2_SO_4_, 2.8 mM MgSO_4_, 0.1% Triton X-100, 0.5 μl of 8 U *Bst *DNA polymerase (New England Biolabs, USA), 40 U of reverse transcriptase (Invitrogen), and 5 μl of target RNA. Six temperatures (60, 61, 62, 63, 64 and 65°C) were screened during RT-LAMP assay, and finally the optimal temperature was monitored by agarose gel electrophoresis. Amplification was completed in PCR microtubes in an electric thermostatic water bath (Suopu, Shanghai, China) at the isothermal condition of 60°C for 60 min. GoldView dye (Sbsgene, China) was used to evaluate the reaction system with the reference of a negative control and a positive control. The samples with fluorescence change from orange to red or from orange to green were considered as the negative and positive reactions under ultraviolet (UV) light (254 nm) with a handheld lamp.

### PCR fluorescence probing assay and nucleotide sequencing

The PCR fluorescence probing assay reagent (DaAn Gene, China) was commercially available. The cDNA was generated in a 20 μl of reaction volume for 25 min at 40°C using random primers and SuperScript II reverse transcriptase (Invitrogen) according to the instructions. The cycling conditions were composed of 5 min at 94°C, followed by 40 cycles with 93°C for 15 s, 55°C for 45 s, and 72°C for 1 min, and a final extension cycle of 72°C for 10 min. The PCR products were examined by Lighter cycler (Roche, Germany). Forward primer EV71-F: 5'-AAA GGT GGA GCT GTT CAC CTA CAT GCG CTT TGA C-3', reverse primer EV71-R: 5'-AAT CTG GCT TGG GGG CCC CAG GTG GTA CAA-3', and oligonucleotide probe EV71-P: 5'-CCC ACC GGG GAA GTT GTC CCA CAA TTG CTC C-3'. The specific oligonucleotide probes were added, and the results were considered as the positive for Ct value not higher than 35.1. All amplicons were sequenced with an ABI 3730 automated DNA sequencer (Applied Biosystems).

### Specificity of RT-LAMP assay

A restriction analysis by using HinfI or TaqI endonuclease was used to validate the specificity of EV71 amplification reaction. Enzymatic reaction system is composed as follows: 2 μl of RT-LAMP product, 15 μl of ddH_2_O, 1 μl of 10 × buffer R, 1 μl of HinfI or TaqI for 10 U/μl (each). The digestion reactions of HinfI or TaqI were incubated at 37°C and 65°C for 2 h, respectively. Finally, 1.5 μl of digested RT-LAMP products were analyzed by agarose gel electrophoresis. Four virus strains of EV71, CVA16, coxsackievirus A4 (CVA4), and coxsackievirus B3 (CVB3) were provided by China Center for Type Culture Collection and Changzhou Center for Disease Control, respectively.

### Sensitivity of RT-LAMP and PCR fluorescence probing assay

The EV71 or CVA16 was propagated in RD cells, and the titer was determined by plaque assay in vero cells [25,26]. Totally 0.5 ml virus culture at various concentrations ranging from 10^5 ^to 0.1 PFU/ml in a serial 10-fold dilution pattern was detected by RT-LAMP and PCR fluorescence probing assay. The products of RT-LAMP were detected by agarose gel electrophoresis.

### EV71 detection in clinical specimens by RT-LAMP and PCR fluorescence probing assay

Totally 93 pharyngeal swabs, 20 vesicular fluid swabs and 10 fecal samples with suspicious EV71 infection were extracted as described above and analyzed by RT-LAMP and PCR fluorescence probing assays with the reference of negative and positive control reactions containing CVA16 and EV71.

### Statistical analysis

Statistically significant analysis of the clinical study was evaluated by using chi-square test. A significant difference was considered at the *P *value of less than 0.05.

## Results

### The optimum reaction system of RT-LAMP assay

**Figure 2:**
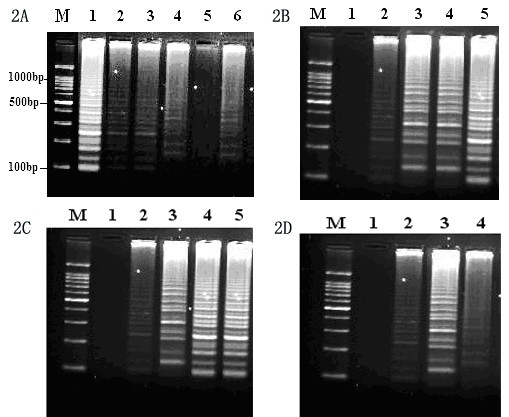
**The optimal condition of RT-LAMP for the detection of EV71**. (2A) The optimal temperature of RT-LAMP assay is monitored by agarose gel analysis. M: Marker, 100-bp DNA ladder (Sigma, USA); Lanes 1-6 are reaction temperatures at 60, 61, 62, 63, 64 and 65°C, respectively. (2B) The reaction time of RT-LAMP. Lanes 1-5 are reaction time of 30, 40, 50, 60 and 70 min, respectively. (2C) The concentration of magnesium. Lanes 1-5 are magnesium at the concentrations of 0, 1, 2, 2.8 and 3.6 mM, respectively. (2D) The concentration of betaine. Lanes 1-4 are the betaine concentrations of 0, 0.5, 1.0 and 1.5 M, respectively.

**Figure 3:**
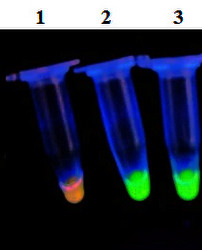
**GoldView staining of the samples**. Samples with the fluorescence change from orange to green under UV lamp at a 254 nm are considered as positive (Lanes 2 and 3), while a sample with fluorescence change from orange to red is considered as negative (Lane 1).

The optimal temperature for RT-LAMP reaction was determined to be 60°C, which could provide the optimal activity of *Bst *DNA polymerase (Figure 2A). Similarly, the optimal reaction time and concentrations of magnesium and betaine were identified as 60 min, 2.8 mM and 1 M, respectively (Figure 2B, C and 2D). Under the GoldView staining, positive samples revealed the fluorescence change from orange to green (Figure 3).

### Restriction enzyme analysis of RT-LAMP products

**Figure 4:**
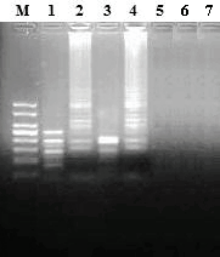
**Agarose gel electrophoresis and restriction analysis of RT-LAMP products**. M: Marker, 100-bp DNA ladder (Sigma, USA); Lanes 1 and 3 are RT-LAMP products digested with HinfI and TaqI, respectively; Lanes 2 and 4 are RT-LAMP amplified products; Lanes 5-7 are CVA16, CVA4 and CVB3 negative controls, respectively.

The specificity of RT-LAMP was confirmed by the digestion using HinfI and TaqI restriction enzymes. The amplified products with EV71 resulted in a series of bands in agarose gel electropherosis. In contrast, non-specific products with CVA16, CVA4 and CVB3 in RT-LAMP assay could not be detected (Figure 4).

### Sensitivity of EV71 virus by RT-LAMP amplification

**Figure 5:**
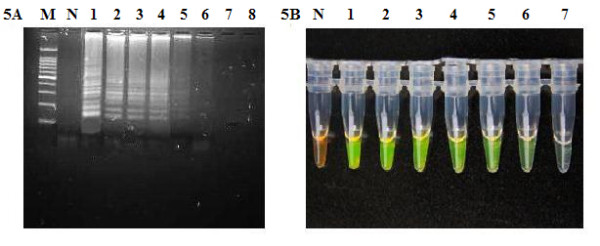
**The detection sensitivity of LAMP assay for EV71**. (5A) The electrophoresis diagram of RT-LAMP with virus serial dilution. M: Marker, 100-bp DNA ladder (Sigma, USA). N: negative control without target DNA. Lanes 1-8 are target DNA at 10^5^, 10^4^, 10^3^, 10^2^, 10, 1, 0.1, and 0 PFU/ml, respectively. (5B) Goldview staining of serial dilution reaction. N: Negative. Lanes 1-7; target DNA at 10^5^, 10^4^, 10^3^, 10^2^, 10, 1 and 0.1 PFU/ml, respectively.

**Figure 6:**
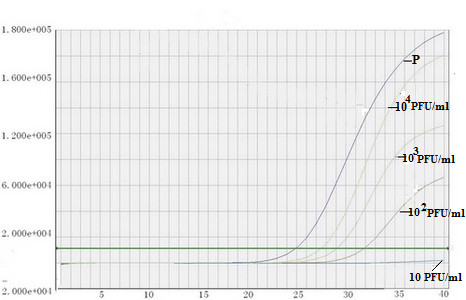
**The detection sensitivity of PCR fluorescence probing assay**. P: EV71 positive control; RNA samples (10^4 ^PFU/ml) with 10^-1^-10^-3 ^dilutions, respectively.

The sensitivity of RT-LAMP and PCR fluorescence probing assay for the detection of EV71 RNA was evaluated by testing serial 10-fold dilutions of virus through previously described as plaque assay. The RT-LAMP assay was able to amplify a small amount of virus in 60 min with a detection limit of 1 PFU/ml of virus (Figure 5A and 5B). RT-LAMP revealed 100-fold higher sensitivity than PCR fluorescence probing assay, which had a detection limit of 100 PFU (copy)/ml of virus (Figure 6).

### Detection of 123 clinical specimens

In order to assess the diagnostic accuracy for suspicious patients with EV71 infection, 123 specimens were subjected to RT-LAMP assay with the parallel analysis by PCR fluorescence probing assay. The amplicons were sequenced. As shown in Table 2, an overall similarity between two test systems was 98.4% (*P >*0.05). No samples with positive reaction detected by PCR fluorescence probing assay was missed during RT-LAMP assay, thereby indicating the superior sensitivity of RT-LAMP. Two positive samples detected by RT-LAMP assay but PCR fluorescence probing assay negative were then inoculated into RD cells and the positive culture was identified by indirect immunofluorescence test; however, only one sample was verified as positive EV71 infection. The positive rates of 93 pharyngeal swabs detected by RT-LAMP and PCR fluorescence probing assay were 44.1 and 41.9%, respectively, and the positive rates of 20 vesicular fluid swabs and 10 fecal samples detected by both methods were 60.0% and 50.0% (Table 2).

**Table 2:** Comparison of 123 specimens from suspicious HFMD patients were analyzed by RT-LAMP and PCR fluorescence probing assay

Specimen type	Number of samples	RT-LAMP		PCR fluorescence probing	
	
		*Positive (%)	#Negative (%)	Positive (%)	Negative (%)
Pharyngeal swabs	93	41 (44.1)	52 (55.9)	39 (41.9)	54 (58.1)
Vesicular fluids	20	12 (60.0)	8 (40.0)	12 (60.0)	8 (40.0)
Fecal samples	10	5 (50.0)	5 (50.0)	5 (50.0)	5 (50.0)
Total	123	58 (47.2)	65 (52.8)	56 (45.5)	67 (54.4)

*Positive: EV71 positive samples; #Negative: EV71 negative samples.

## Discussion

Human enterovirus belongs to the RNA virus family *Picornaviridae *including polioviruses, type A coxsackieviruses, type B coxsackieviruses, echoviruses, and enterovirus types 68 to 71 (EV68-71) [15,27]. Due to the prevalence of EV71 infection, time-consuming viral culture and serological tests for EV71, it is important to develop fast, efficient and sensitive diagnostic reagents [28,29]. Although PCR technique has been widely used in the detection of infectious diseases and the real-time PCR assay has multiple advantages such as faster quantitative measurement, lower contamination rate, higher sensitivity, higher specificity and easier standardization, these nucleic acid amplification methods require high-precision instruments and skilled technicians [30,31].

LAMP is a nucleic acid amplification method that relies on autocycling strand displacement DNA synthesis completed by *Bst *DNA polymerase. Because of high sensitivity and specificity coupled with the requirements for only a water bath and endpoint detection using a color-change reaction visible with the naked eye [32-34], LAMP assay has been applied for the analysis of various infections from *Mycobacterium tuberculosis, Norovirus, Human papillomavirus, Cytomegalovirus*, and *Human immunodeficiency virus *[20,35-38].

In this study, a one-step and simple assay for EV71 virus without requirement of thermal cycling can be completed within 60 min in a single tube containing the mixture of buffer, primers, reverse transcriptase and *Bst *DNA polymerase. In order to ensure its accuracy and reliable amplification, it is very important to optimize the reaction temperature. At the optimal reaction temperature of 60°C, clear bands of amplified products were observed in gel electrophoresis.

Similarly, the optimal reaction time, magnesium and betaine concentrations were achieved. Except for amplified products detected by agarose gel electrophoresis as described above, two alternative detection methods could also be used for the evaluation. 1) *Turbidity*: the accumulation of magnesium pyrophosphate, a by-product of amplified DNA, results in the increase of the turbidity in samples. The turbidity in samples was evaluated by visual inspection. However, when the virus content of samples is low, it is difficult to observe the turbidity by visual inspection. 2) *Fluorescence change*: GoldView staining was used for evaluating the amplification. Samples with the fluorescence conversion from orange to green or from orange to red under an UV handheld lamp at a 254 nm were considered as the positive and negative, respectively. Different from traditional ethidium bromide (EB) for detecting double-stranded DNA, single-stranded DNA, and RNA, Goldview staining can emit green fluorescence when bound to dsDNA and red fluorescence when bound to ssDNA or RNA. However, compared to EB with strong mutagen, Goldview has relatively fewer mutations and higher safety.

Particularly, similar clinical symptoms in infections caused by EV71 and CVA16 with high similarity in nucleotide sequences were observed. Therefore, frequent mis-diagnosis between EV71 and CVA16 infections was happened due to the difficulty in distinguishing both viruses [39]. However, exact nucleotide sequences of RT-LAMP products can be predicted from target DNA and primers. Thus, it is possible to predict the digestion outcome of the samples with restriction enzymes at the specific recognition sites. In the present study, the specific products could be digested by HinfI and TaqI restriction enzymes to result in a series of bands in agarose gel electrophoresis, in contrast, non-specific products could not be detected due to without digestion. Therefore, RT-LAMP assay can provide high specificity for the detection of EV71.

The sensitivity of RT-LAMP assay for EV71 detection was determined by testing serial 10-fold dilutions of virus with a detection limit of 1 PFU/ml. In order to evaluate the reliability of RT-LAMP, 123 suspicious samples were simultaneously detected by PCR fluorescence probing assay and RT-LAMP. Both test systems had 98.4% similarity without significant difference. With a detection limit of 1 PFU/ml, RT-LAMP assay exhibited much higher sensitivity than PCR fluorescence probing assay, as well as virus culture. Between two positive samples identified by RT-LAMP but PCR fluorescence probing assay negative, only one sample was verified as EV71 infection by virus culture and indirect immunofluorescence test, which further confirmed the higher sensitivity of RT-LAMP assay.

Besides pharyngeal, vesicular fluid and stool, RT-LAMP can be used to detect other samples such as cerebrospinal fluid, etc. However, the application of RT-LAMP in these samples needs to be further explored to provide more evidences in the early diagnosis of EV71 infection.

## Conclusions

RT-LAMP assay is characteristics of high sensitivity, rapid detection, high specificity and low cost, which has considerable potentials for the detection of EV71 infection in the primary health care institutions, field environments or developing countries.

## Competing interests

The authors declare that they have no competing interests.

## Authors' contributions

WS conceived the study, carried out the molecular genetic studies, analyzed the data and drafted the manuscript; KL and YJ participated in the design and carrying out specimen collection; QJ performed PCR-Fluorescence probing assay, agar gel electrophoresis and analyzed the data; MS and ZM helped coordinate the investigation and participated in LAMP assay. All authors contributed to the study and have read and approved the final manuscript.

## Pre-publication history

The pre-publication history for this paper can be accessed here:

http://www.biomedcentral.com/1471-2334/11/197/prepub
